# Prophylactic Effect of *Lactobacillus fermentum* TKSN02 on Gastric Injury Induced by Hydrochloric Acid/Ethanol in Mice Through Its Antioxidant Capacity

**DOI:** 10.3389/fnut.2022.840566

**Published:** 2022-03-01

**Authors:** Tiantian Hu, Liang Zhou, Xiaoli Wang, Xianrong Zhou, Ruokun Yi, Xingyao Long, Xin Zhao

**Affiliations:** ^1^Chongqing Engineering Laboratory for Research and Development of Functional Food, Chongqing Collaborative Innovation Center for Functional Food, Chongqing Engineering Research Center of Functional Food, Chongqing University of Education, Chongqing, China; ^2^TCM (Rheumatic Immunology/Geriatrics) Department, People's Hospital of Chongqing Banan District, Chongqing, China

**Keywords:** *Lactobacillus fermentum*, gastric injury, ethanol, oxidation, probiotic

## Abstract

In this article, the preventive and protective effect of a new *Lactobacillus fermentum*, (*Lactobacillus fermentum* TKSN02: LF-N2), which was isolated and identified from Xinjiang naturally fermented yogurt, on hydrochloric acid (HCl)/ethanol induced gastric injury in mice was studied. A total of 40 mice were divided into the following five groups: normal, model, LF-N2, LB (*Lactobacillus bulgaricus*), and Ranitidine groups. Except for the normal and model groups, mice in the other groups were treated with LF-N2, LB (*Lactobacillus* bulgaricus), and Ranitidine separately, and the injury of the gastric tissue was observed by taking photos and pathological sections. The levels of oxidation indicators, gastrointestinal hormone and the inflammatory cytokines in serum and gastric tissue in each group were measured. Further more, the gene expression levels of oxidative stress and inflammation related genes in the colon tissue were determined by the Real-Time PCR method. Pathological observation confirmed that LF-N2 could inhibit the gastric injury caused by HCl/ethanol. Observation of the appearance of the gastric indicated that LF-N2 could effectively reduce the area of gastric injury. Biochemical results showed that the serum gastrin (GAS) and gastric motilin (MTL) levels in the LF-N2 group were significantly lower and the serum somatostatin (SS) level was higher than in the model group and there was no significant difference between all treatment groups. The activities of total superoxide dismutase (T-SOD) and glutathione (GSH) were increased while the malondialdehyde (MDA) content was decreased in LF-N2 treatment group mice, which suggested that LF-N2 has a good antioxidant effect. Further RT-PCR experiments also showed that LF-N2 could promote the related mRNA expression of antioxidant enzymes (Cu/Zn-SOD, Mn-SOD, and CAT) and anti-inflammatory cytokines (IL-4, and IL-10), while it inhibited the gene expression of pro-inflammatory cytokine (IL-6) and apoptosis factor (Caspase-3). As observed, LF-N2 exerted a good preventive effect on HCl/ethanol induced gastric injury in mice, and the effect was close to that of LB, which indicated that LF-N2 has potential use as a probiotic due to its gastric injury treatment effects.

## Introduction

According to the World Health Organization (1) 2014 global alcohol and health report, 5.1% of the global burden of disease is attributed to alcohol consumption. In recent years, an increasing number of diseases have been caused by alcohol, among which alcohol-induced gastric injury is particularly prominent. Mucosal injury, chronic gastritis, and gastric ulcer are the important manifestations of gastric injury (2, 3). When alcohol, acid, irritant substances, and some drugs achieve a certain concentration or dose, they can disrupt the gastric environmental homeostasis and the barrier function of gastric mucosa itself, and induce gastric injury (4). Stomach injury can lead to obvious gastrointestinal abnormalities. The clinical manifestations are abdominal pain, loss of appetite, nausea and vomiting, abdominal distension and belching, intragastric bleeding, and hematemesis. In addition, long-term and regular consumption of alcoholic beverages has been considered as a risk factor for causes of various cancers, especially for increasing the risk of gastric cancer (5, 6).

Inhibition of gastric acid secretion and protection of gastric mucosa are the main methods to prevent and treat alcohol-induced gastric mucosal injury. The protective mechanism involves blocking the expression of inflammatory factors, reducing the inflammatory response, and preventing the signal transduction of gastric mucosal lesions (5). Currently, most of the drugs available in the market for the treatment of gastric ulcer, such as ranitidine and omeprazole, have an obvious curative effect. However, long-term use of such drugs can cause side effects and adverse reactions (7). Therefore, it is necessary to develop a more effective and safer anti-ulcer functional drug food.

Probiotics are living microorganisms that can adjust a variety of physiological functions by affecting the flora of the digestive tract. When administered in adequate amounts, probiotics are beneficial to the health of the host. The beneficial effect of probiotics has been widely explored in the study of gastrointestinal diseases. Probiotics have been proved to exert preventive and protective effects on intestinal inflammation and mucosal damage caused due to various reasons (8–12). Currently, there are many studies on the preventive and protective effects of probiotics on gastric injury, including the good effect of probiotics in the prevention and treatment of Helicobacter pylori related gastropathy and the preventive and protective effect on gastric injury caused by non-steroidal anti-inflammatory and analgesic drugs (NSAIDs), such as aspirin, acetic acid, and ethanol. It has potential research value in alcohol-induced gastric injury (13–15).

Due to great differences in the health functions of different strains of probiotics, the strains found in traditional fermented food in various countries have their own advantages and are considered to have research prospects. Xinjiang, located in the southwest of China, has a favorable climate and rich dairy products since ancient times. Through long-term selection and inheritance, naturally fermented yogurt has achieved high nutritional value and unique flavor, and it has become one of the favorite foods of local residents. It is naturally fermented by lactic acid bacteria (LAB), and its yield is far more than that of fresh milk.

A new *Lactobacillus fermentum*, named *L. fermentum* TKSN2 (LF-N2), was isolated from the yogurt in Yili Prefecture, Xinjiang. In this study, a mouse model of gastric injury was established by the interaction of alcohol and hydrochloric acid (HCl). The gastric- protective potential of LF-N2 in the mouse model of gastric injury induced by HCl/ethanol solution was studied by taking *Lactobacillus* isolated from Xinjiang naturally fermented yogurt as the research object, ranitidine as the positive drug control, and *Lactobacillus bulgaricus* (LB) as the comparative strain. Compared with LB, this strain of *Lactobacillus* has great potential.

## Materials and Methods

### Preparation of *Lactobacillus fermentans*

*Lactobacillus fermentum* TKSN02 used in this study was isolated and identified from the traditional fermented yak yogurt samples obtained from herdsmen in Kuoketiereke Township, Tekes County, Yili Prefecture, Xinjiang, China. The preservation number was CGMCC No. 18221 in China General Microbiological Culture Collection Center, Beijing, China (hereafter referred to as LF-N2). *Lactobacillus bulgaricus* was purchased from the Institute of Microbiology of the Chinese Academy of Sciences, Beijing, China (*delbrueckii subsp. Bulgaricus*: CGMCC No. 1.16075, hereafter referred to as LB). After activation on two occasions, frozen LF-N2 and LB were counted, and the volume of bacterial suspension solution was prepared by determining the concentration and number of bacteria required for gavage administration.

### Determination the Hydrophobic Property

A sample of 5 mL reactivated bacterial was centrifuged and resuspend in 5 mL PBS (phosphate buffer) buffer (50 mM, pH 6.5), and then the suspension was centrifuged at 3,000 rpm for 10 min; the process was repeated once again. PBS was used as the blank of absorption, the final bacteria suspension was adjusted by PBS buffer to make a 1.00 absorbance (denoted as A0) at 560 nm. In total 4 mL of the bacteria suspension was mixed with 0.8 mL of dimethylbenzene, vibrated for 30 s and then placed for stratification. The aqueous layer was measured for the absorbance (denoted as A) at 560 nm (blank: PBS buffer) and the results were recorded.

### Tolerance to Artificial Gastric Juice

Artificial gastric juice consists of 0.2% NaCl and 0.35% pepsin, and then 1 mol/L of HCl was added to adjust the pH of the prepared artificial gastric juice to 3.0. In a survey, 0.22 μm filter membrane was used to filter the artificial gastric juice to sterilize and set aside. Resuspend the centrifuged bacterial fluid with equal volume of sterile saline, and then 1 ml of bacterial solution was mixed with 9 ml of artificial gastric juice. One mL of the above mixture was taken as a sample for 0 h, and the remaining 9 mL of mix was placed in a constant temperature water bath shaker (37 °C, 150 r/min) for 3 h. The samples of 0 h and 3 h were diluted by 10 times gradient, the appropriate gradient was selected by the method of plate coating to determine the number of live bacteria, and the survival rate (%) was calculated according to The following formula:

survival rate (%) = 3-h viable count (CFU/mL)/0-h viable count (CFU/mL) × 100.

### Resistance to Bile Salts

In total, 0.3% pig bile salt solution was added to MRS-THIO medium (MRS medium containing 0.2% sodium thiol acetate) to its final concentration of 0.3%. Then, 5 mL of the activated strain was inoculated with 2% (v/v) inoculation volume into MRS-THIO media without and with 0.3% bile salt separately, followed by incubation at 37°C for 24 h. The optical density (OD) of the medium was determined at 600 nm. The tolerance to bile salts was calculated based on the following formula: bile salts tolerance (%) = (OD of 0.3% bile salts medium – OD of the blank medium)/(OD of 0.0% bile salts medium – OD of the blank medium) × 100.

### Detection of DPPH Radical Scavenging Ability

The DPPH radical scavenging ability of LF-N2 was measured by using 0.2 mM DPPH solution with absolute ethanol. Equal volume of the test sample (1 mL) and DPPH solution were thoroughly mixed and incubated in dark for 30 min at room temperature, and then centrifuged at 6,000 rpm for 10 min. The supernatant was taken and the absorbance at 517 nm was measured, namely A1. The absorbance value, A2, was measured with anhydrous ethanol instead of DPPH solution. Sterile distilled water was used instead of the sample solution and the absorbance value, A3, was measured. DPPH clearance rate was calculated according to the formula: DPPH clearance (%) = (1-(A1-A2)/A3) × 100.

### Animal Experiment

Forty C57BL/6 mice (SPF grade, male, aged 5 weeks) were purchased from Chongqing Medical University and adaptively fed in an environment at constant temperature and humidity for a week. Forty mice were randomly divided into the following five groups (*n* = 8): Normal group, Model group, LF-N2 group, LB group, and Ranitidine group. Mice in the normal and model groups were treated with normal saline once a day by gavage for 14 days (0.1 mL/10 g), while mice in the bacterial intervention group were treated with LF-N2 or LB liquid (1.0 × 10^9^ CFU/kg) by gavage for 14 days. Mice in the drug treatment group were given ranitidine (200 mg/kg) by gavage every day. On the 14th day after the end of gavage, the mice were fasted for 24 h but allowed to drink freely. Excluding the normal group, the other groups were treated with a mixture of 60% ethanol and 40% 150 mmol/mL HCl by gavage at a dose of 0.1 mL/10 g according to the weight of mice once (16).

All mice were exposed to CO_2_ for 30 min after gastric injury was induced. The blood was taken from the ocular venous plexus by eyeball extraction, and then the serum was obtained by centrifugation (4,000 rpm for 10 min at 4°C and the supernatant stored at −80°C), and other tissues were collected for fixation and cryopreservation.

### Histopathological Observation of the Stomach

The gastric tissue of mice was placed on the test bench, laid out, and photographed and then tissue measuring about 0.5 cm in length and width was cut for paraffin section and hematoxylin and eosin (H&E) staining. The gastric tissue were immersed in 10% formalin fixation solution for 48 h, followed by dehydration, embedding in paraffin, sectioning with a thickness of 5–8 μm, and staining with H&E, and finally photographed under a microscope with different magnification (Olympus, Tokyo, Japan).

### Detection of Oxidative Stress Factors in Serum and Gastric Tissue

Blood samples were collected by enucleating eyeball method, and then they were centrifuged at 4,000 rpm for 10 min at 4°C to obtain serum. The serum was aspirated and saved standby at −80°C for future use. The contents of malondialdehyde (MDA) and glutathione (GSH) in serum, the activity of total superoxide dismutase (T-SOD), and the content of MDA in the gastric tissue homogenate of mice were determined according to the instructions of the respective kit (Solarbio Life Sciences, Beijing, China).

### Detection of Gastrointestinal Hormones and Inflammatory Factors in Serum and Gastric Tissue

Serum levels of gastrin (GAS), somatostatin (SS) and interleukin-4 (IL-4), interleukin-6 (IL-6), and interleukin-10 (IL-10) were measured according to the instructions of ELISA kit. Further, 50 mg gastric tissue was homogenized (50 mg gastric tissue and 4–6 magnetic beads are put into the homogenization tube. The parameters of the homogenizer were set to 60 M/s, 30 sec pause/30 sec vibration and 6 cycles. After homogenization, the homogenization solution was transferred to a new centrifugal tube for use), and then the level of MTL in the gastric tissue homogenate was determined according to the instructions of ELISA kit (Solarbio Life Sciences, Beijing, China).

### Detection of mRNA Expression

Total RNA was extracted from 50 to 100 mg gastric tissue of each sample using TRIzol reagent (Invitrogen, Carlsbad, CA, USA). Then, the RNA solution was diluted to 1 μg/μL after the concentration and purity of RNA were detected. RNA was reverse-transcribed into cDNA following the reverse transcription kit instructions (Tiangen Biotech Co., Ltd., Beijing, China). Finally, the target gene was amplified by real-time quantitative polymerase chain reaction using the gene primers listed in Table 1.

**Table 1:** Sequences of gene primers used in qPCR.

**Gene name**	**Sequence**
*CAT*	Forward: 5′-GGAGGCGGGAACCCAATAG-3′
	Reverse: 5′-GTGTGCCATCTCGTCAGTGAA-3′
*Caspase-3*	Forward: 5'-TCTGACTGGAAAGCCGAAAC-3'
	Reverse: 5'-GCAAGCCATCTCCTCATCAG-3'
*Mn-SOD*	Forward: 5'-CAGACCTGCCTTACGACTATGG-3'
	Reverse: 5'-CTCGGTGGCGTTGAGATTGTT-3'
*Cu/Zn-SOD*	Forward: 5′-AACCAGTTGTGTTGTCAGGAC-3′
	Reverse: 5′-CCACCATGTTTCTTAGAGTGAGG-3′
*eNOS*	Forward: 5′-TCAGCCATCACAGTGTTCCC-3′
	Reverse: 5′-ATAGCCCGCATAGCGTATCAG-3′
*nNOS*	Forward: 5′-GAATACCAGCCTGATCCATGGAA-3′
	Reverse: 5′-TCCTCCAGGAGGGTGTCCACCGCATG-3′
*GAPDH*	Forward: 5′-AGGTCGGTGTGAACGGATTTG-3′
	Reverse: 5′-GGGGTCGTTGATGGCAACA-3′

### Statistical Analysis

All data were expressed as mean ± standard deviation. Differences between individual groups were assessed by one-way analysis of variance (ANOVA) with Duncan's multiple range test. *P* < 0.05 indicated a statistically significant difference.

## Results

### *In vitro* Screening of LF-N2 Activities

By the *in vitro* experiments, the survival rate of LF-N2 in the artificial gastric juice at pH 3.0 and in 0.3% bile salts was 91.24 ± 2.11% and 11.62 ± 1.35%, and the hydrophobic property was 14.05 ± 1.22%, the result showed that the strain was highly resistant to artificial gastric juice. The DPPH free radical scavenging rates of LF-N2 complete cells and non-cell extracts were 58.2 ± 8.3% and 94.79 ± 18.4%, respectively, which showed strong *in vitro* antioxidant properties.

### Macroscopic Observation of the Stomach

**Figure 1:**
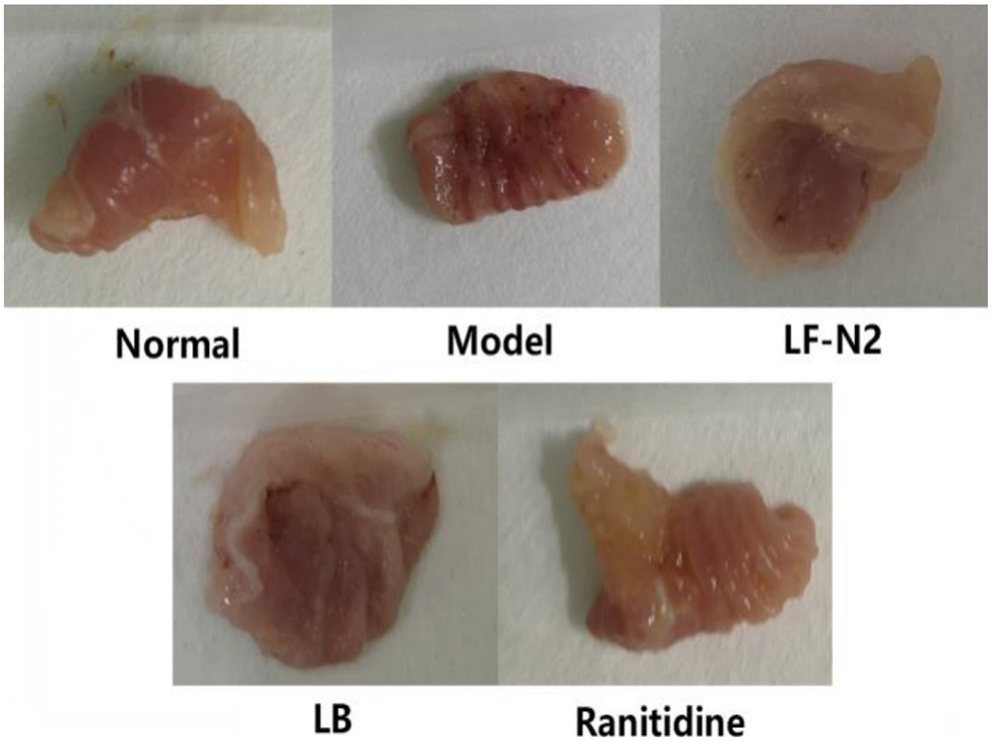
Observation of gastric mucosa in mice. LF-N2, *Lactobacillus fermentum* TKSN02 (1 × 10^9^ CFU//kg); LB, *Lactobacillus bulgaricus* (1 × 10^9^ CFU/kg); ranitidine (200 μg/g).

After the HCl/ethanol gavage intervention, injuries in mouse stomachs were observed by stomach photographs (Figure 1). The stomachs of mice in the normal group showed no injury and the surface of gastric mucosa was clean and smooth with vivid color. However, there were obviously many bleeding spots and small area injuries in the stomach of mice in the model group. Compared with the model group, the gastric tissue of mice in the LF-N2 and LB treatment groups showed no obvious damage, and the color tended to be normal, leaving only a few bleeding points. The ranitidine group was not different from the normal group. Calculation revealed that the inhibition of LF-N2 in gastric injury was highly effective, which was close to that of ranitidine and LB.

### Pathological Observation of Gastric Tissue

**Figure 2:**
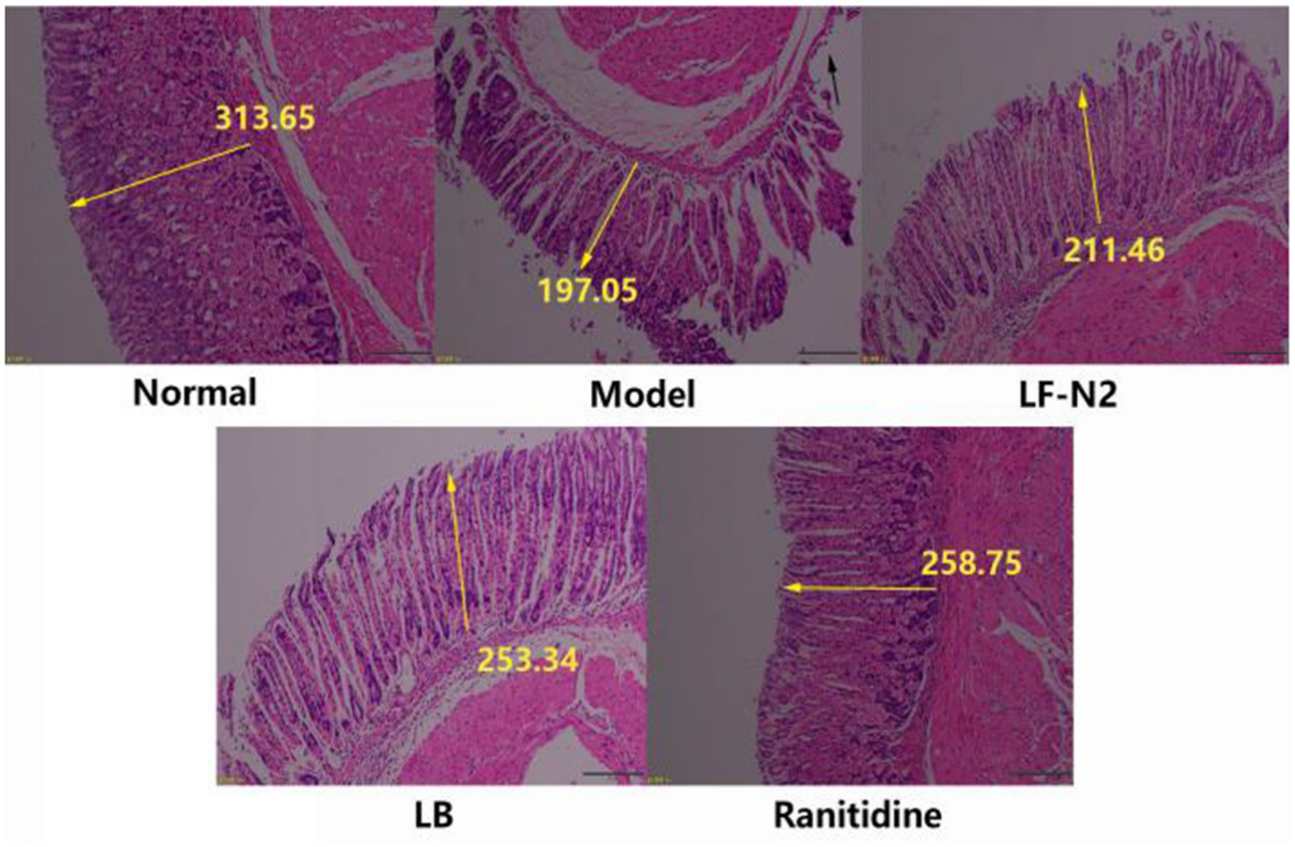
Levels of antioxidant factors in serum and gastric tissue. LF-N2, *Lactobacillus fermentum* TKSN02 (1 × 10^9^ CFU//kg); LB, *Lactobacillus bulgaricus* (1 × 10^9^ CFU/kg); ranitidine (200 μg/g). The yellow arrow shows the relative length.

Pathological changes in gastric tissue were observed under a light microscope. As shown in Figure 2, the structure of gastric mucosa in normal mice was complete, there was almost no rupture or damage, the glands were arranged in an orderly manner, the surface epithelium was intact, and there was no inflammatory cell infiltration in the lamina propria mucus layer, which showed that the gastric mucosa was not be damaged by intragastric administration of normal saline. Compared with the normal group, the model group showed severe rupture and abscission of the upper epidermis of gastric mucosa. And the thickness of gastric mucosa became thinner (see yellow arrow mark), the loose connective tissue in the submucosa became wider, and there might be some edema. In addition, inflammatory cell infiltration could be seen, indicating successful establishment of a model of gastric mucosal injury. Gastric mucosa showed different degrees of pathological damage in the three treatment groups, but it was better than that in the model group, similar to ranitidine, which had a good inhibitory effect on gastric mucosal injury, and the epithelial cells of gastric mucosa were nearly complete, the glands were arranged in an orderly manner, and the secretion of inflammatory cells was decreased. The above results of histological analysis showed that pretreatment with LF-N2 could reduce (HCl)/ethanol induced histological damage of the gastric mucosa surface and protect the gastric tissue.

### Levels of Antioxidant Factors in Serum and Gastric Tissue

**Figure 3:**
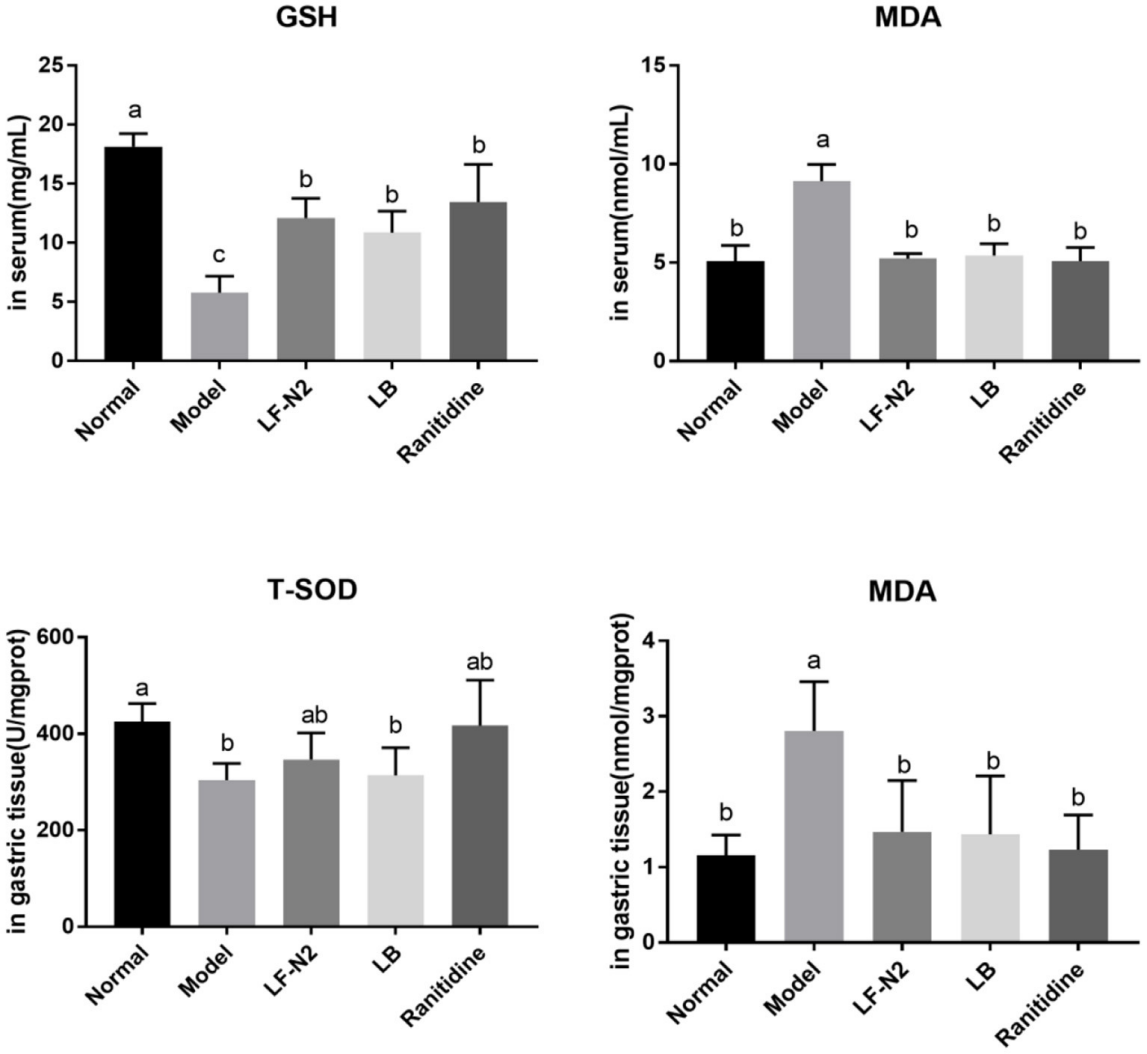
Levels of antioxidant factors in serum and gastric tissue, (*n* = 6). LF-N2, *Lactobacillus fermentum* TKSN02 (1 × 10^9^ CFU//kg); LB, *Lactobacillus bulgaricus* (1 × 10^9^ CFU/kg); ranitidine (200 μg/g). ^a−*c*^Mean values with different letters in the same bars are significantly different (*P* < 0.05).

As seen in Figure 3, compared with the normal group, the serum GSH content and gastric T-SOD activity in the model group were significantly decreased, while the MDA content was significantly increased in model group. While compared with the model group, the levels of T-SOD and GSH in the three treatment intervention groups were significantly increased (*P* < 0.05), and the content of MDA was significantly decreased (*P* < 0.05). In general, it seems that the effect of the ranitidine group was the best, and the indexes of serum and gastric tissue were significantly better than those in the model group, LB Group, and LF-N2 group, which were equivalent to those in the control group, but there was no significant difference between the three treatment groups.

### Levels of GAS, SS, and MTL in Serum and Gastric Tissue

**Figure 4:**
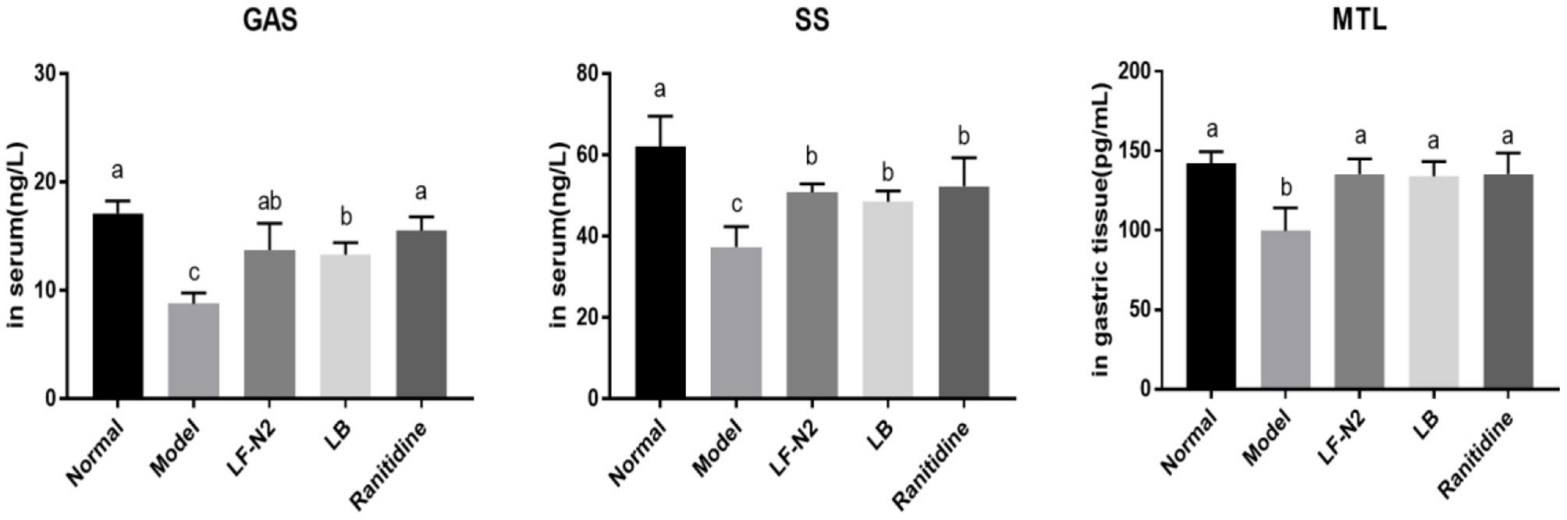
Levels of GAS, SS and MTL in serum and gastric tissue, (*n* = 6). LF-N2, *Lactobacillus fermentum* TKSN02 (1 × 10^9^ CFU//kg); LB, *Lactobacillus bulgaricus* (1 × 10^9^ CFU/kg); ranitidine (200 μg/g). ^a−*c*^Mean values with different letters in the same bars are significantly different (*P* < 0.05).

The serum GAS and MTL levels in mice in the normal group were the lowest, but the serum SS levels in these mice were the highest (Figure 4). LF-N2, LB, and ranitidine treatments significantly (*P* < 0.05) reduced the serum GAS and MTL levels and raised the serum SS levels compared with those in control mice. Also, there was no significant difference among all treatment groups.

### Levels of Inflammatory Cytokines IL-4, IL-6, and IL-10 in Serum

**Figure 5:**
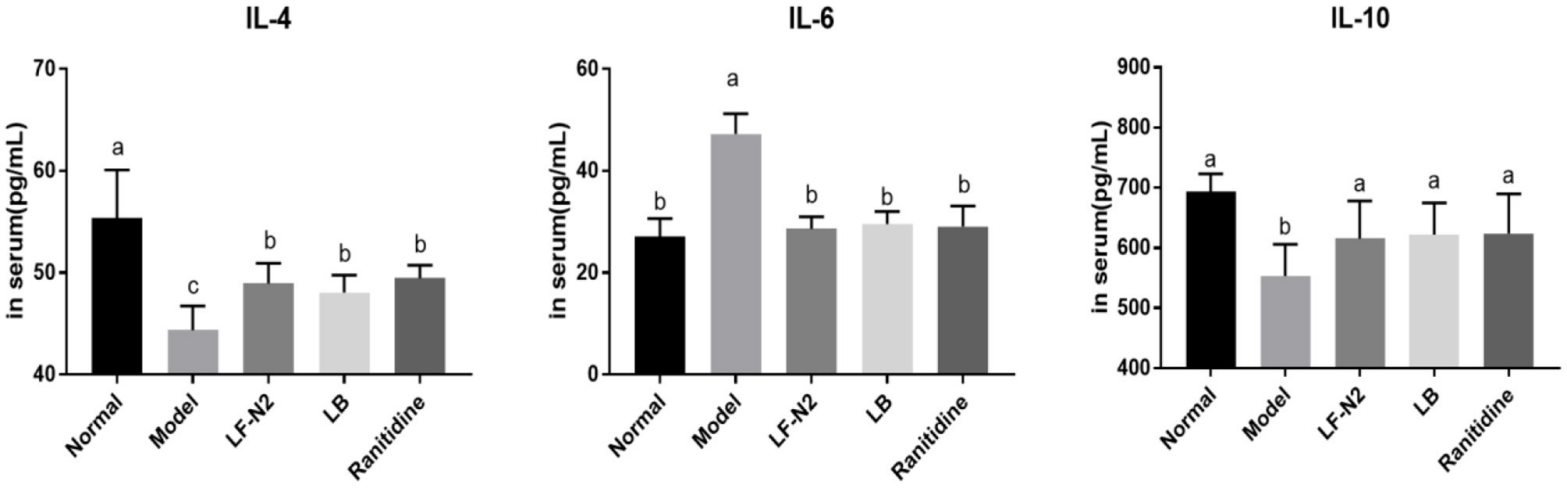
Inflammatory cytokines levels of IL-4, IL-6 and IL-10 in serum, (*n* = 6). LF-N2, *Lactobacillus fermentum* TKSN02 (1 × 10^9^ CFU//kg); LB, *Lactobacillus bulgaricus* (1 × 10^9^ CFU/kg); ranitidine (200 μg/g). ^a−*c*^Mean values with different letters in the same bars are significantly different (*P* < 0.05).

As seen from the results in Figure 5, compared with the normal group, IL-4 and IL-10 levels in the model group were decreased significantly, while the IL-6 level was increased. The expression of IL-6 was effectively reduced, and the expressions of IL-4 and IL-10 were increased in the three treatment groups. Although the three treatments could reverse the expression of inflammatory factors in the model group, there was no significant difference among the groups.

### Expression Levels of Gastric Corpus Related Genes in Mice

**Figure 6:**
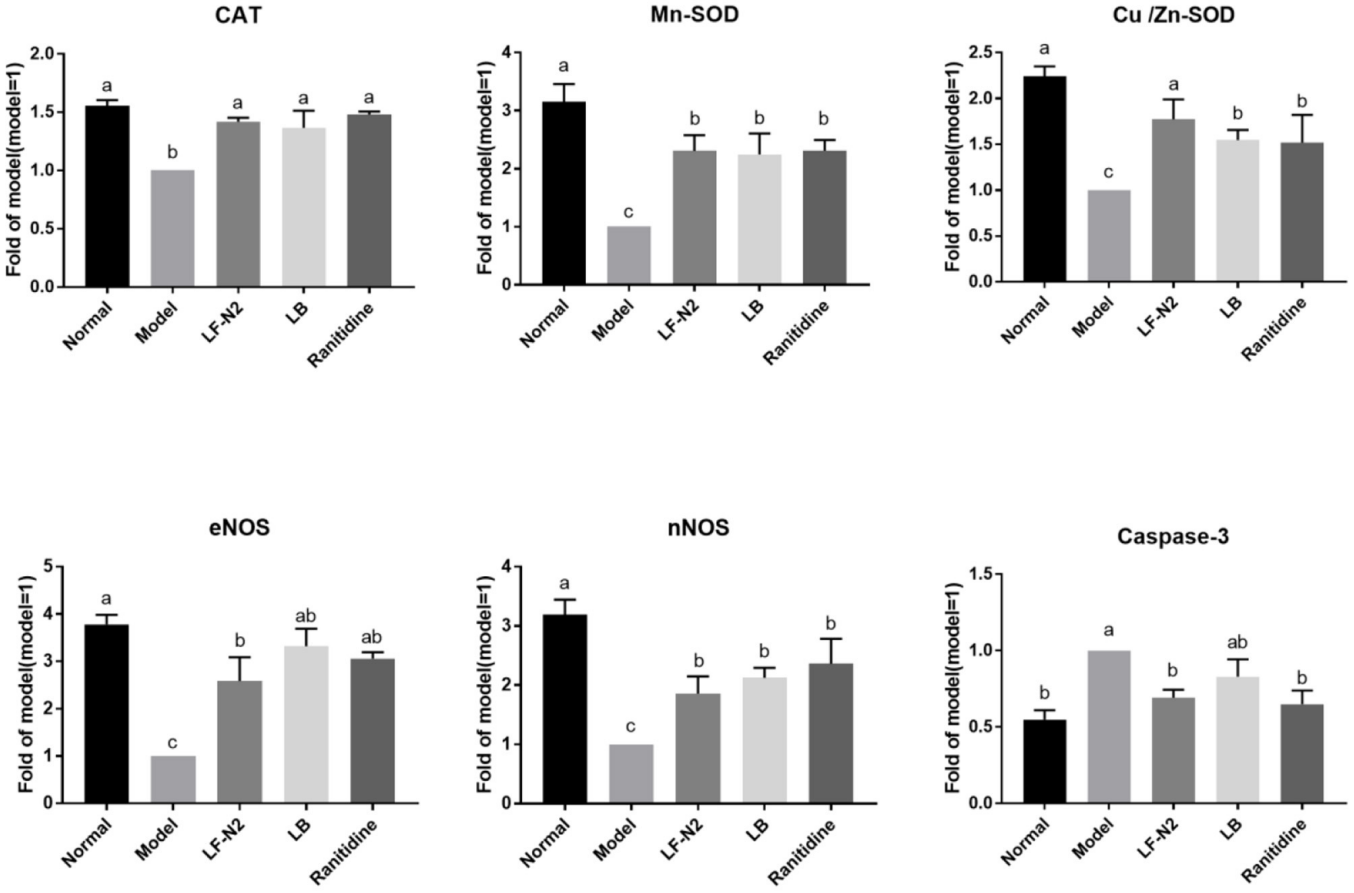
mRNA expression in gastric tissue of mice with with HCl/ethanol-induced gastric injury. LF-N2, *Lactobacillus fermentum* TKSN02 (1 × 10^9^ CFU//kg); LB, *Lactobacillus bulgaricus* (1 × 10^9^ CFU/kg); ranitidine (200 μg/g). ^a−*c*^Mean values with different letters in the same bars are significantly different (*P* < 0.05).

The expression of several genes (Figure 6) in the gastric corpus of mice were analyzed to understand the effects of administration of live *Lactobacillus fermentans* TKSN02. The results showed that the mRNA expressions of CAT, Mn-SOD, Cu/Zn-SOD, eNOS, and nNOS were significantly decreased (*P* < 0.05) and that of caspase-3 was increased (*P* < 0.05) in the model group compared with the normal group. However, compared with the model group, treatment with LF-N2 could significantly reverse the expression trend of the above genes, which improved the expressions of CAT, Mn-SOD, Cu/Zn-SOD, eNOS, and nNOS genes and reduced the expression of caspase-3 (*P* < 0.05). And there was little difference between the three treatment groups (*P* > 0.05).

## Discussion

The combination of alcohol and HCl can aggravate injury to the gastric mucosa. Gastric injury can lead to different degrees of damage to other tissues and systems, such as the blood system, digestive system, and nervous system. Long-term gastric tissue injury and inflammation are also risk factors of gastric cancer; hence, early prevention and treatment of gastric injury is very important. It has been reported that some probiotics have beneficial effects on alcohol-induced gastric injury in animal models and clinical trials (8–10, 12). For example, related studies have shown that *Lactobacillus acidophilus* can effectively alleviate the symptoms of gastric mucosal injury in mice and inhibit the weight loss in mice (17). In this study, the model of gastric injury induced by alcohol and HCl was used to verify the potential of the new *Lactobacillus fermentum* TKSN02 (LF-N2) isolated and identified from Xinjiang natural fermented yogurt in the prevention and protection of gastric injury induced by HCl/ethanol in mice, and its potential mechanism was preliminarily studied.

First, the potential probiotic functions of LF-N2 were identified by measuring the acid-resistance, cholate tolerance and hydrophobic property. LF-N2 showed high qualities in above activities *in vitro* and even better acid-resistance than LB, which could result in funcrional effects for use in animals or humans (18). At the same time, the good antioxidant effect of LF-N2 *in vitro* showed that this strain may has a high potential for use *in vivo*. Further in animal experiment, after intragastric administration of alcohol and HCl, a large area of bleeding point and visible damage of gastric tissue were observed; compared with the model group, the gastric injury in LF-N2, LB, and ranitidine intervention groups was significantly reduced. Furthermore, the pathological section confirmed that LF-N2 could inhibit the ethanol/HCl induced gastric tissue injury in mice and effectively reduce the area of ethanol/HCl induced gastric injury.

The mechanism of gastric mucosal injury induced by HCl/alcohol may involve excessive gastric acid secretion, and a decrease in the pH of gastric juice in mice aggravates the gastric mucosal injury (19). When ethanol induced gastric injury causes bleeding, the blood flow to the gastric mucosa decreases, sodium^+^ is pumped out, and K^+^ is pumped in, resulting in a significant increase in gastric acid secretion. Therefore, reducing gastric acid secretion is the main prevention and treatment measure of ethanol/HCl induced gastric mucosal injury (20). In addition, it also includes the disorder of gastric mucosa microorganisms, oxidative damage caused by excessive production of oxygen free radicals, and inflammatory reaction caused by excessive production of nitric oxide (NO) (8).

First, several types of GAS related to gastric acid secretion were detected. GAS and MTL belong to the GAS family, which can coordinate the contraction of esophagus and gastric sphincter, promote the secretion of gastric acid and pancreatic juice, and regulate gastrointestinal peristalsis. GAS is a typical digestive hormone and growth factor, which is mainly secreted by gastric mucosal G cells. Its function is to regulate gastric acid secretion and control the growth of gastric mucosal cells. A large number of studies have shown that GAS peptide and its receptor are related to the occurrence of cancer. SS is another important component of digestive juice, which can inhibit GAS release and gastric acid secretion.

After gastric mucosal injury, the secretion of GAS and MTL was increased, which further led to an increase in gastric acid secretion, thus reducing pH and aggravating the severity of acute gastric mucosal injury. Research has also reported that acute stomach injury caused by alcohol reduces the SS levels *in vivo* (21). The biochemical results in this study showed that serum GAS and gastric MTL levels in the LF-N2 group were significantly lower and the serum SS level was higher than the corresponding levels in the model group, and there was no significant difference among all treatment groups. GAS is induced by nutrients and GAS releasing peptides. On the contrary, SS inhibits GAS release at a high concentration of H^+^. This suggests that LF-N2 treatment may downregulate GAS content and finally regulate gastric acid secretion by promoting SS secretion.

An important inducement of gastric mucosal injury is oxidative damage caused by excessive oxygen free radicals caused by external stimulation. Ethanol forms acetaldehyde under the catalysis of ethanol dehydrogenase in the stomach, which is further catalyzed by xanthine oxidase to produce free radicals, resulting in oxidative damage (22). MDA is a lipid peroxide, which can reflect the level of oxygen free radicals in organisms. In aerobic organisms, T-SOD is a key scavenger of reactive oxygen species, which can remove superoxide anion free radicals, protect cells from damage, and play a vital role in the balance between oxidation and antioxidation (23, 24). GSH is a non-protein compound and a thiol containing compound. As an important antioxidant and free radical scavenger in the body, GSH can eliminate free radicals and play a strong antioxidant role (25). It is necessary to detect the activity of antioxidant enzymes to judge the ability of drugs or probiotics to scavenge oxygen free radicals, and to detect the level of lipid peroxidation product MDA to judge the severity of oxidative damage of tissues and cells attacked by free radicals. The result obtained in this study showed that LF-N2 increased the activities of T-SOD and GSH, while it decreased the MDA content in mice, which suggested that it has a good antioxidant effect. In addition, the expression of antioxidant enzyme genes, such as CAT and SOD were further detected. SOD is a general term of the SOD family, and in vertebrates, SOD is primarily expressed as Cu/Zn-SOD and Mn-SOD (26). Similar to SOD, CAT is an important antioxidant enzymes. Free radicals can be converted into less toxic H_2_O_2_ by SOD, and then they can be converted into H_2_O by CAT, thereby scavenging free radicals (27). In this study, Cu/Zn-SOD, Mn-SOD, and CAT mRNA expressions in the three intervention groups were significantly higher than those in the model group. So far, the strains that have been proved to have antioxidant capacity include: *Lactobacillus acidophilus, Lactobacillus sake, Bifidobacterium longum, Lactobacillus fermentum*, etc (28–32).

Uncontrolled drinking and gastric injury will activate the immune system, resulting in changes in the level of inflammation and inflammatory factors. IL-6 is a proinflammatory factor, which is widely involved in inflammation, and it may induce neutrophil aggregation, lead to neutrophil respiratory burst, form reactive oxygen species, and lead to tissue inflammation (26). However, it has been proved that IL-4 and IL-10 belong to the family of anti-inflammatory cytokines, which can down regulate IL-8 produced by LPS activated human polymorphonuclear cells (PMN), and IL-10 is considered to be the most active anti-inflammatory cytokine (33). IL-10 has been considered to have potent broad-spectrum anti-inflammatory activity, which has been clearly confirmed in various infection, inflammation, and cancer models (34). Nitric oxide (NO) is an important signaling molecule in cells, which has a protective effect on gastric mucosa and can increase the function of gastric mucosa. cNOS (eNOS and nNOS) is a type of key enzyme in NO biosynthesis. A study has shown that the cNOS activity decreases significantly after gastric mucosal injury (35). An increase in the contents of eNOS and nNOS can effectively prevent and alleviate gastric ulcer symptom (36, 37). In this study, LF-N2 significantly inhibited IL-6, increased the levels of IL-4 and IL-10 in serum, and the gene expression of NOS in gastric tissue, thereby inhibiting gastric inflammation and injury.

This study showed that LF-N2 can prevent acute gastric injury by regulating the expression of antioxidant markers, pro-inflammatory cytokines and anti-inflammatory cytokines, and can be used to develop functional food. Although the mechanism of LF-N2 is not clear, we speculate that it may be related to Nrf2-ARE signaling pathway. Nrf2-ARE system is considered as a multi organ protection system. Transcription factor Nrf2 plays an indispensable role in the induction of endogenous antioxidant enzymes (38, 39). Under oxidative stress or injury conditions, Nrf2 is released from the keap1-Nrf2 complex and translocated to the nucleus, where There, the Nrf2/Maf complex is formed and the Nrf2 are pathway is activated (40). Nrf2/Maf complex can not only activate the dependent gene expression of a series of antioxidant and cytoprotective proteins, but also play a physiological role through its anti-inflammatory, antioxidant, detoxification, autophagy and proteasome effects (41). SOD1 is a downstream molecule of Nrf2, the nuclear accumulation of Nrf2 can increase the expression of SOD1 and Keap1/Nrf2/ARE regulates glutathione (GSH) levels by upregulating GSH synthetic and regenerative enzymes (42). In addition, Nrf2 can reverse regulate NF-κB-driven inflammatory response, in the brain injury model, Nrf2-KO mice produced higher levels of IL-6 than wild-type mice (43, 44). In addition to the aspects mentioned above, the mechanism of gastric protective effect of LF-N2 may be related to the interaction between bacterial products and immune system. LAB and their components can activate macrophages, NK cells and B lymphocytes and other immune cells. Macrophages may internalize and secrete TNF- α, NO, IFN-γ, IL-2, IL-6, IL-8, IL-12 and IL-18, so as to play an anti-inflammatory and even anti-tumor role. LAB peptidoglycan and lipoteichoic acid are inducers of NOS, the cell walls of various LAB can stimulate mouse macrophages and other immune cells to produce NO and play an anti-inflammatory effect by combining with oxygen to form highly lethal hydroxyl free radicals and NO_2_. However, the research on the mechanism of LF-N2 is still in the early stage of exploration, and it can be further explored from the Nrf2/ARE signal pathway in the future.

## Conclusion

A new LAB was isolated from traditional Xinjiang natural fermented yogurt and named *Lactobacillus fermentum* TKSN02 (LF-N2). After studying LF-N2 in the mouse model of HCl/ethanol-induced gastric injury, we found that it not only improves HCl/ethanol-induced gastric tissue injury (such as histological damage of gastric mucosa surface and gastric bleeding), but it also acts at the serum and mRNA levels, by regulating the expression of antioxidant markers, pro-inflammatory cytokines, and anti-inflammatory cytokines. We propose that LF-N2 is a useful probiotic strain with the potential to confer gastro-protection and alleviate acute gastric injury. Other potential probiotic effects and further clinical trials of LF-N2 treatment are necessary to determine whether such treatment could benefit human patients with gastric problems.

## Data Availability Statement

The original contributions presented in the study are included in the article/supplementary material, further inquiries can be directed to the corresponding author/s.

## Ethics Statement

The animal study was reviewed and approved by the Ethics Committee of Chongqing Medical University, (202011001B, Chongqing, China) and it was in accordance with the national standard of the People's Republic of China (GB/T 35892–2018) laboratory animal guidelines for ethical review of animal welfare.

## Author Contributions

TH and LZ are mainly responsible for the content of the experiment and manuscript writing. XW, XZ, RY, and XL are mainly involved in data analysis research. XZ oversaw the research and reviewed the final manuscript. All authors contributed to the article and approved the submitted version.

## Funding

This research project has been supported by the Construction of Double Cities Economic Circle in Chengdu-Chongqing Region Science and Technology Innovation Project (KJCX2020052), General Program of Natural Science Foundation of Chongqing (cstc2021jcyj-msxmX0408), and Chongqing University Innovation Research Group Project (CXQTP20033), China.

## Conflict of Interest

The authors declare that the research was conducted in the absence of any commercial or financial relationships that could be construed as a potential conflict of interest.

## Publisher's Note

All claims expressed in this article are solely those of the authors and do not necessarily represent those of their affiliated organizations, or those of the publisher, the editors and the reviewers. Any product that may be evaluated in this article, or claim that may be made by its manufacturer, is not guaranteed or endorsed by the publisher.

## References

[1] Organization WH. Global status report on alcohol and health. Global Status Report Alcohol. (2014) 18:1–57.

[2] Lee E Lee JE. Impact of drinking alcohol on gut microbiota: recent perspectives on ethanol and alcoholic beverage. Curr Opin Food Sci. (2021) 37:91–7. 10.1016/j.cofs.2020.10.001

[3] Wang XX Yin AW Tao ZP Ying LI Gao PF. Research progress on anti-alcoholic gastric injury active components and mechanisms of Chinese herbal medicine. China J Chin Materia Medica. (2020) 45:4836–45. 10.19540/j.cnki.cjcmm.20200917.609 33350254

[4] Na HK Lee JY. Molecular basis of alcohol-related gastric and colon cancer. Int J Mol Sci. (2017) 18:1116–32. 10.3390/ijms18061116 28538665 PMC5485940

[5] Dong J Thrift AP. Alcohol, smoking and risk of oesophago-gastric cancer. Best Pract Res Clin Gastroenterol. (2017) 31:509–17. 10.1016/j.bpg.2017.09.002 29195670

[6] The L. Alcohol and cancer. Lancet. (2017) 390:2215. 10.1016/S0140-6736(17)32868-4 29165257

[7] Sontag SJ. Guilty as charged: Bugs and drugs in gastric ulcer. Am J Gastroenterol. (1997) 92:1255–61. 9260785

[8] Gomi A Harima-Mizusawa N Shibahara-Sone H Kano M Miyazaki K Ishikawa F. Effect of Bifidobacterium bifidum BF-1 on gastric protection and mucin production in an acute gastric injury rat model. J Dairy Sci. (2013) 96:832–7. 10.3168/jds.2012-5950 23200466

[9] Senol A Isler M Karahan AG Kilic GB Kuleasan H Kaya S. Preventive effect of probiotics and alpha-tocopherol on ethanol-induced gastric mucosal injury in rats. J Med Food. (2011) 14:173–9. 10.1089/jmf.2010.0040 21244242

[10] Suo H Zhao X Qian Y Sun P Zhu K Li J. *Lactobacillus fermentum* Suo attenuates HCl/Ethanol induced gastric injury in mice through its antioxidant effects. Nutrients. (2016) 8:155. 10.3390/nu8030155 26978395 PMC4808883

[11] Lam EK Yu L Wong HP Wu WK Shin VY Tai EK. Probiotic *Lactobacillus rhamnosus* GG enhances gastric ulcer healing in rats. Eur J Pharmacol. (2007) 565:171–9. 10.1016/j.ejphar.2007.02.050 17395175

[12] Wang R Zeng X Liu B Yi R Zhou X Mu J. Prophylactic effect of *Lactobacillus plantarum* KSFY06 on HCl/ethanol-induced gastric injury in mice. Food Funct. (2020) 11:2679–92. 10.1039/C9FO02474C 32162630

[13] Akama F Nishino R Makino S Kobayashi K Kamikaseda K Nagano J. The effect of probiotics on gastric mucosal permeability in humans administered with aspirin. Scand J Gastroenterol. (2011) 46:831–6. 10.3109/00365521.2011.574730 21492054

[14] Gao F Sui L Mu G Qian F Zhu X. Screening of potential probiotics with anti- Helicobacter pylori activity from infant feces through principal component analysis. Food Biosci. (2021) 42:101045–55. 10.1016/j.fbio.2021.101045

[15] Kakiuchi T Mizoe A Yamamoto K Imamura I Matsuo M. Effect of probiotics during vonoprazan-ontaining triple therapy on gut microbiota in Helicobacter pylori infection: a randomized controlled trial. Helicobacter. (2020) 25:177–86. 10.1111/hel.12690 32207209

[16] Alrashdi AS Salama SM Alkiyumi SS Abdulla MA Hadi AH Abdelwahab SI. Mechanisms of gastroprotective effects of ethanolic leaf extract of jasminum sambac against HCl/Ethanol-induced gastric mucosal injury in rats. Evid Compl Alternat Med. (2012) 2012:786426–41. 10.1155/2012/786426 22550543 PMC3329065

[17] Wagner RD Warner T Roberts L Farmer J Dohnalek M Hilty M. Variable biotherapeutic effects of *Lactobacillus acidophilus* isolates on orogastric and systemic candidiasis in immunodeficient mice. Rev Iberoam Micol. (1998) 15:271–6. 18473516

[18] Zhao X Qian Y Suo HY Du MY LI GJ Liu ZH. Preventive effect of lactobacillus fermentum zhao on activated carbon-induced constipation in mice. J Nutr Sci Vitaminol. (2015) 61:131–7. 10.3177/jnsv.61.131 26052143

[19] Laine L Takeuchi K Tarnawski A. Gastric mucosal defense and cytoprotection: bench to bedside. Gastroenterology. (2008) 135:41–60. 10.1053/j.gastro.2008.05.030 18549814

[20] Olguin-Martinez M Hernandez-Espinosa DR Hernandez-Munoz R. High alpha-tocopherol dosing increases lipid metabolism by changing redox state in damaged rat gastric mucosa and liver after ethanol treatment. Clin Sci. (2018) 132:1257–72. 10.1042/CS20180154 29773670

[21] Zhou YL Wang R Feng X Zhao X. Preventive effect of insect tea against reserpine-induced gastric ulcers in mice. Exp Ther Med. (2014) 8:1318–24. 10.3892/etm.2014.1859 25187847 PMC4151677

[22] Raish M Ahmad A Ansari MA Alkharfy KM Aljenoobi FI Jan BL. Momordica charantia polysaccharides ameliorate oxidative stress, inflammation, and apoptosis in ethanol-induced gastritis in mucosa through NF-kB signaling pathway inhibition. Int J Biol Macromol. (2018) 111:193–9. 10.1016/j.ijbiomac.2018.01.008 29307809

[23] Sun J Wen X Liu J Kan J Qian C Wu C. Protective effect of an arabinogalactan from black soybean against carbon tetrachloride-induced acute liver injury in mice. Int J Biol Macromol. (2018) 117:659–64. 10.1016/j.ijbiomac.2018.05.203 29852225

[24] Miller AF. Superoxide dismutases: ancient enzymes and new insights. FEBS Lett. (2012) 586:585–95. 10.1016/j.febslet.2011.10.048 22079668 PMC5443681

[25] Chang W Bai J Tian S Ma M Li W Yin Y. Autophagy protects gastric mucosal epithelial cells from ethanol-induced oxidative damage via mTOR signaling pathway. Exp Biol Med. (2017) 242:1025–33. 10.1177/1535370216686221 28056554 PMC5444638

[26] Wang YW Zheng GY Chen XC Zhang J Pan XD. Inhibitory action of tripchlorolide on activation of gliacyte and p38MAPK induced by Aβ25-35 injected into hippocampus of rats. Chin Pharmacol Bul. (2014) 30:108–13. 10.3969/j.issn.1001-1978.2014.01.024

[27] Kiruthiga PV Shafreen RB Pandian SK Arun S Govindu S Devi KP. Protective effect of silymarin on erythrocyte haemolysate against benzo(a)pyrene and exogenous reactive oxygen species (H2O2) induced oxidative stress. Chemosphere. (2007) 68:1511–8. 10.1016/j.chemosphere.2007.03.015 17481694

[28] Gan Y Chen X Yi R Zhao X. Antioxidative and anti-inflammatory effects of *Lactobacillus plantarum* ZS62 on alcohol-induced subacute hepatic damage. Oxid Med Cell Longev. (2021) 2021:7337988. 10.1155/2021/7337988 34912498 PMC8668337

[29] Gan Y Tong J Zhou X Long X Pan Y Liu W. Hepatoprotective effect of *Lactobacillus plantarum* HFY09 on ethanol-induced liver injury in mice. Front Nutr. (2021) 8:684588. 10.3389/fnut.2021.684588 34249992 PMC8264191

[30] Liu J Tan F Liu X Yi R Zhao X. Grape skin fermentation by *Lactobacillus fermentum* CQPC04 has anti-oxidative effects on human embryonic kidney cells and apoptosis-promoting effects on human hepatoma cells. RSC Adv. (2020) 10:4607–20. 10.1039/C9RA09863A PMC9049054 35495273

[31] Pan Y Wang H Tan F Yi R Li W Long X. *Lactobacillus plantarum* KFY02 enhances the prevention of CCl4-induced liver injury by transforming geniposide into genipin to increase the antioxidant capacity of mice. J Funct Foods. (2020) 73:104128. 10.1016/j.jff.2020.104128

[32] Amanatidou A Smid EJ Bennik MH Gorris LG. Antioxidative properties of *Lactobacillus sake* upon exposure to elevated oxygen concentrations. FEMS Microbiol Lett. (2001) 203:87–94. 10.1111/j.1574-6968.2001.tb10825.x 11557145

[33] Marie C Pitton C Fitting C Cavaillon JM. Regulation by anti-inflammatory cytokines (IL-4, IL-10, IL-13, TGFβ) of interleukin-8 production by LPS- and/ or TNFα-activated human polymorphonuclear cells. Mediat Inflam. (1996) 5:334–40. 10.1155/S0962935196000488 18475727 PMC2365804

[34] Mosser DM Xia Z. Interleukin-10: new perspectives on an old cytokine. Immunol Rev. (2010) 226:205–18. 10.1111/j.1600-065X.2008.00706.x 19161426 PMC2724982

[35] Gong J Zhang Z Zhang X Chen F Tan Y Li H. Effects and possible mechanisms of Alpinia officinarum ethanol extract on indomethacin-induced gastric injury in rats. Pharm Biol. (2018) 56:294–301. 10.1080/13880209.2018.1450426 29781354 PMC6130516

[36] Chen W Wu D Jin Y Li Q Liu Y Qiao X. Pre-protective effect of polysaccharides purified from Hericium erinaceus against ethanol-induced gastric mucosal injury in rats. Int J Biol Macromol. (2020) 159:948–56. 10.1016/j.ijbiomac.2020.05.163 32450327

[37] Wang XY Yin JY Zhao MM Liu SY Nie SP Xie MY. Gastroprotective activity of polysaccharide from Hericium erinaceus against ethanol-induced gastric mucosal lesion and pylorus ligation-induced gastric ulcer, and its antioxidant activities. Carbohydr Polym. (2018) 186:100–9. 10.1016/j.carbpol.2018.01.004 29455967

[38] Wang J Fields J Zhao C Langer J Thimmulappa RK Kensler TW. Role of Nrf2 in protection against intracerebral hemorrhage injury in mice. Free Radic Biol Med. (2007) 43:408–14. 10.1016/j.freeradbiomed.2007.04.020 17602956 PMC2039918

[39] Lan X Han X Li Q Wang J. (-)-Epicatechin, a natural flavonoid compound, protects astrocytes against hemoglobin toxicity via Nrf2 and AP-1 signaling pathways. Mol Neurobiol. (2017) 54:7898–907. 10.1007/s12035-016-0271-y 27864733 PMC5436959

[40] Cheng D Wu R Guo Y Kong ANT. Regulation of Keap1-Nrf2 signaling: The role of epigenetics. Curr Opin Toxicol. (2016) 1:134–8. 10.1016/j.cotox.2016.10.008 29057383 PMC5645054

[41] Tu W Wang H Li S Liu Q Sha H. The Anti-Inflammatory and anti-oxidant mechanisms of the Keap1/Nrf2/ARE signaling pathway in chronic diseases. Aging Dis. (2019) 10:637–51. 10.14336/AD.2018.0513 31165007 PMC6538222

[42] Stefanson AL Bakovic M. Dietary regulation of Keap1/Nrf2/ARE pathway: focus on plant-derived compounds and trace minerals. Nutrients. (2014) 6:3777–801. 10.3390/nu6093777 25244368 PMC4179188

[43] Bellezza I Grottelli S Gatticchi L Mierla AL Minelli A. alpha-Tocopheryl succinate pre-treatment attenuates quinone toxicity in prostate cancer PC3 cells. Gene. (2014) 539:1–7. 10.1016/j.gene.2014.02.009 24530478

[44] Bellezza I Mierla AL Minelli A. Nrf2 and NF-kappaB and their concerted modulation in cancer pathogenesis and progression. Cancers. (2010) 2:483–97. 10.3390/cancers2020483 24281078 PMC3835087

